# Comparative Efficacy of Hemagglutinin, Nucleoprotein, and Matrix 2 Protein Gene-Based Vaccination against H5N1 Influenza in Mouse and Ferret

**DOI:** 10.1371/journal.pone.0009812

**Published:** 2010-03-23

**Authors:** Srinivas S. Rao, Wing-Pui Kong, Chih-Jen Wei, Neal Van Hoeven, J. Patrick Gorres, Martha Nason, Hanne Andersen, Terrence M. Tumpey, Gary J. Nabel

**Affiliations:** 1 Vaccine Research Center, National Institute of Allergy and Infectious Diseases (NIAID), National Institutes of Health, Bethesda, Maryland, United States of America; 2 Influenza Division, Centers for Disease Control and Prevention, Atlanta, Georgia, United States of America; 3 Biostatistics Research Branch, National Institute of Allergy and Infectious Diseases (NIAID), National Institutes of Health, Bethesda, Maryland, United States of America; 4 BIOQUAL, Inc., Rockville, Maryland, United States of America; Statens Serum Institute, Denmark

## Abstract

Efforts to develop a broadly protective vaccine against the highly pathogenic avian influenza A (HPAI) H5N1 virus have focused on highly conserved influenza gene products. The viral nucleoprotein (NP) and ion channel matrix protein (M2) are highly conserved among different strains and various influenza A subtypes. Here, we investigate the relative efficacy of NP and M2 compared to HA in protecting against HPAI H5N1 virus. In mice, previous studies have shown that vaccination with NP and M2 in recombinant DNA and/or adenovirus vectors or with adjuvants confers protection against lethal challenge in the absence of HA. However, we find that the protective efficacy of NP and M2 diminishes as the virulence and dose of the challenge virus are increased. To explore this question in a model relevant to human disease, ferrets were immunized with DNA/rAd5 vaccines encoding NP, M2, HA, NP+M2 or HA+NP+M2. Only HA or HA+NP+M2 vaccination conferred protection against a stringent virus challenge. Therefore, while gene-based vaccination with NP and M2 may provide moderate levels of protection against low challenge doses, it is insufficient to confer protective immunity against high challenge doses of H5N1 in ferrets. These immunogens may require combinatorial vaccination with HA, which confers protection even against very high doses of lethal viral challenge.

## Introduction

Since 1997, the highly pathogenic avian influenza A H5N1 viral strain has caused severe disease in poultry and wild birds. Although H5N1 has not spread widely in humans, sporadic infections have been seen throughout countries of eastern Asia, the Middle East and Africa. To date, there have been more than 445 confirmed human cases of H5N1, with 263 deaths (59% mortality rate) reported by the World Health Organization (http://www.who.int/csr/disease/avian_influenza/country/cases_table_2009_12_11/en/index.html). In almost all cases, those infected with H5N1 had physical contact with infected birds. While the primary mode of transmission may be animal-to-human, the concern remains that this virus may evolve into a strain capable of human-to-human transmission. Vaccination offers a practical and effective measure for controlling the spread of this highly pathogenic virus. The threat posed by emerging strains of influenza is unpredictable and varies among countries, as evidenced by the recent swine origin H1N1 pandemic, highlighting the need for improved vaccines that can confer broad protection against multiple viral strains and various influenza A subtypes.

While the hemagglutinin (HA) surface protein is conventionally the primary target of strain-specific influenza DNA vaccines, conserved viral epitopes have the potential to induce immunity against diverse influenza strains. Two highly conserved influenza viral proteins, NP and M2, have been widely targeted as possible broadly protective vaccine candidates [1]–[9]. The main function of the nucleoprotein is encapsidation of the viral genome to form a ribonucleoprotein particle for transcription and packaging. NP also interacts with other viral proteins (PB1, PB2, and M1) and cellular proteins (Importin α, F-actin, CRM1/exportin1) for viral transcription control and nuclear transportation control [10]. M2 is responsible for protein translocation, and is expressed at a high density in the plasma membrane of infected cells in tetramer forms. This ion channel protein is also a target for the antiviral drugs amantidine and rimantadine, which control viral replication and have been used for influenza prophylaxis and treatment [11].

In mice, DNA/rAd5 vaccination with NP and M2 from the H1N1 PR/8 strain induced both humoral and cellular immune responses that protected against lethal H5N1 challenges [12]. However, the mouse model is not ideal for the evaluation of H5N1 infection and vaccines due to differences in HA receptor specificity and distribution, influenza pathogenicity, as well as clinical symptomatology [13]–[17]. Infection in the ferret shows greater similarity to human infection in terms of anatomical distribution and disease. Outbred ferrets exhibit severe lethargy, fever, weight loss, and transient lymphopenia, as well as viral replication in the upper and lower respiratory tract, brain, and multiple systemic organs after infection with various strains of H5N1 virus. Thus, this model is widely considered to be more reflective of human influenza infection [13], [18], [19]. While we continued to evaluate the protective efficacy of NP and M2 in the mouse model, we extended our investigation into the ferret model in this study.

Previous studies have investigated the protective efficacy of these conserved epitopes against lethal influenza challenge in mice and ferrets [7], [20], [21]. DNA vaccination with NP in combination with M2 formulated with Vaxfectin has been shown to protect mice against heterosubtypic challenge with H3N2 and H1N1 viruses [7]. Vaxfectin-formulated DNA vaccine encoding NP+M2 protected mice, but against high challenge doses of H5N1 virus in ferrets it only delayed time to illness and death [20]. However, triple prime with rAd boost regimens of NP in combination with M2 have been shown to protect ferrets against the H5N1 virus, albeit at a relatively low challenge dose [21]. In this study, we evaluated protective immunity induced by NP and M2 alone or in combination with HA in a triple prime, rAd boost regimen against high dose lethal H5N1 challenge. Initially, we tested the ability of DNA immunization with HA alone, NP alone, HA+NP, and HA+NP+M2 to protect against high doses of lethal H5N1 challenge in mice. We then assessed the ability of NP and M2, alone or in combination, to protect ferrets against a high challenge dose of lethal H5N1 virus, when delivered in a triple DNA prime, rAd5 boost regimen. We compared these groups to ferrets immunized with HA alone or in combination with NP+M2.

## Materials and Methods

### Immunogen and plasmid construction

Plasmids encoding HA (A/Thailand/1(KAN-1)/2004, GenBank AY555150), NP (A/Thailand/1(KAN-1)/2004, GenBank AAV35112 and A/PR/8/34, GenBank AAM75159), and M2 (A/Thailand/1(KAN-1)/2004, GenBank AAV35111) were synthesized using human-preferred codons and constructed in a CMV/R backbone by GeneArt (Regensburg, Germany) as previously described [22]. Gene expression was confirmed using 293T (Invitrogen, Carlsbad, CA) transfected cells by Western blot analysis.

### Adenovirus production

Three separate replication-defective rAd serotype 5 vectors expressing HA (KAN-1), NP (KAN-1), and M2 (KAN-1) genes were produced as previously described [23]. Briefly, the genes were inserted into the GV11 adenoviral vector system (GenVec, Gaithersburg, MD), which is based on human serotype 5 and contains deletions of the E1 and E4 regions and part of the E3 region, rendering it replication-defective. The vectors used were as described elsewhere [23], [24]. The vector stocks were serially passaged in complementing mammalian cells (293-ORF6) to generate high-titer stocks of replication-defective adenoviruses [25], [26]. Absence of replication-competent adenovirus was confirmed by product-release assays. Gene expression in A549 (American Type Culture Collection, Manassas, VA) cells was confirmed by Western blot analysis.

### Expression of immunogens in cell culture

Prior to animal immunization, plasmids and adenoviruses encoding various influenza viral genes were tested for their expression in 293T and A549 cells. Plasmids encoding HA protein of A/Thailand/1/KAN-1/2004, NP proteins of A/PR/8/34 and A/Thailand/1/KAN-1/2004, and M2 protein of A/Thailand/1/KAN-1/2004 were transferred into 293T cells using the calcium phosphate-mediated ProFection® Mammalian Transfection system (Promega, Madison, WI). Adenoviruses encoding HA (KAN-1), NP (KAN-1) and M2 (KAN-1) were transfected into A549 cells for 48 hours followed by a change of media. Cell lysates were collected 48 hours post-transfection and subjected to Western blot analysis by mouse monoclonal antibodies against HA (KAN-1), NP (KAN-1), NP (PR/8), and by ferret anti-serum raised against M2 (KAN-1). Specific bands of the predicted size of proteins were detected by comparison to a known vector control.

### H5N1 virus production for viral challenge

The highly virulent A/Vietnam/1203/2004 (H5N1) virus, isolated from a human with a fatal case of influenza [19], was used in this study. The virus stock was propagated in the allantoic cavities of 10-day-old embryonated hens' eggs following incubation at 37°C for 24 to 29 hours. Allantoic fluid from multiple eggs was pooled, clarified by centrifugation, divided into aliquots, and stored at −70°C. The 50% egg infectious dose (EID_50_) titers were determined by serial titration of viruses in eggs and calculated by the Reed and Muench method [27]. The 50% lethal dose (LD_50_) was determined as previously described [19]. All research with HPAI virus was conducted under Biosafety Level 3 containment, including enhancements required by the U.S. Department of Agriculture and the Select Agent Program [28].

### DNA vaccination and viral challenge of mice

All animal research was conducted under the guidance of the Centers for Disease Control and Prevention's Institutional Animal Care and Use Committee in an animal facility accredited by the Association for Assessment and Accreditation of Laboratory Animal Care International. Female BALB/c mice, 6–8 weeks old (Jackson Labs, Bar Harbor, ME), were immunized as previously described [22] with HA from A/Thailand/1(KAN-1)/2004, NP from A/PR/8/34, and M2 from A/Thailand/1(KAN-1)/2004. Briefly, mice (10 animals for each group of HA alone, HA+NP, and HA+NP+M2; and 5 animals for the NP alone and vector control) were immunized three times with a total of 15 µg plasmid DNA in 100 µl of PBS (pH 7.4) intramuscularly at 0, 3, and 6 weeks. For the single HA or NP plasmid group, each group received 5 µg DNA for each plasmid with 10 µg of control vector as filler DNA (total 15 µg). For the 2 plasmid combination group (HA+NP), 5 µg of each plasmid plus 5 µg control vector was used. For the 3 plasmid combination group (HA+NP+M2), 5 µg of each plasmid DNA was used as total DNA remained the same (15 µg). Serum was collected 10 days after the last DNA vaccination.

Viral challenge experiments were performed 3 weeks after the last immunization. All challenged animals were exposed under CO_2_ anesthesia to an intranasal viral inoculum of 100 LD_50_ of A/Vietnam/1203/2004 virus. Body weight and survival were monitored for 21 days. The viral challenge experiments were conducted at the Centers for Disease Control and Prevention (Atlanta, GA) as previously described [17].

### DNA and rAd5 vaccination and viral challenge of ferrets

Male Fitch ferrets, 6–12 months of age (Triple F Farms, Sayre, PA), that were serologically negative by hemagglutinin inhibition (HAI) assay for currently circulating influenza viruses, were used in this study. All HA, NP and M2 genes are from A/Thailand/1(KAN-1)/2004. The numbers of animals used in our studies were as follows: (a) 4 animals for each group of NP and M2 alone, (b) 5 animals for the NP+M2 and the negative control group, (c) in another experiment, 4 animals for all three groups: HA alone, HA+NP+M2 and the vector control. The ferrets were immunized three times with a total of 250 µg plasmid DNA in 500 µl of PBS (pH 7.4) intramuscularly in the quadriceps muscle at 0, 3, and 6 weeks. For the single component plasmid group, each animal received 83 µg DNA for each plasmid with 167 µg of control vector as filler DNA (total 250 µg). For the 2 plasmid combination group (e.g. NP+M2), 83 µg of each NP and M2 plasmid with 83 µg control vector was used (total 250 µg). For the 3 plasmid combination group (HA+NP+M2), 83 µg of each three plasmid DNA was used as total DNA remained the same (total 250 µg). At week 9, the ferrets were immunized intramuscularly with 10^10^ particles of recombinant adenoviruses expressing HA, NP, M2, or in different combinations similar to their DNA immunization combinations. Serum was collected 10 days after the last vaccination. The DNA and adenovirus immunizations were conducted at BioQual, Inc. (Rockville, MD). Nine to ten weeks after the adenovirus boost, the immunized ferrets were challenged with A/Vietnam/1203/2004 virus, which has the identical NP and M2 amino acid sequence as that of the immunized strain A/Thailand/1(KAN-1)/2004, at the Centers for Disease Control and Prevention (Atlanta, GA) as previously described [19]. Briefly, 2 days prior to infection, baseline serum, body temperature, and weight measurements of the ferrets were obtained. After the ferrets were anesthetized by an intramuscular injection of ketamine hydrochloride (24 mg/kg), xylazine (2 mg/kg) and atropine (0.05 mg/kg) cocktail, they were inoculated intranasally with 10^7^ EID_50_ of virus in 1 ml of PBS. The ferrets were monitored for changes in body temperature and weight and the presence of the following clinical symptoms: sneezing, lethargy, anorexia, nasal or ocular discharge, dyspnea, diarrhea, and neurological dysfunction. Body temperatures were measured using an implantable subcutaneous temperature transponder (BioMedic Data Systems, Inc., Seaford, DE). Viral titers were measured in nasal washes collected on days 3, 5, and 7 post-infection from anesthetized ferrets as previously described [6]. The nasal washes were immediately frozen on dry ice and stored at −70°C until they were processed. Viral titers in the nasal washes were determined in eggs as described above. Any ferret that lost more than 25% of its body weight or exhibited neurological dysfunction was euthanized and submitted to postmortem examination. Body weight, clinical symptoms, signs of morbidity, and survival were monitored for 7 or up to 13 days. The statistical significance of differences in survival between groups was determined using a log-rank test.

### Enzyme-linked immunosorbent assay measuring humoral responses of mice and ferret sera

The ELISA assay used in this study was previously described in detail [22], [29], [30]. Briefly, ELISA plates were coated with antigens (100 µl/well) of the following:

- for anti-HA titer: purified HA (KAN-1) (1 µg/ml) as previously described [31],
- for anti-NP (PR/8) titer: purified NP (PR8) (1 µg/ml; Imgenex, San Diego, CA),
- for anti-NP (KAN-1) titer: 1∶3 dilution of the supernatant of NP (KAN-1) encoded plasmid transfected 293 cells,
- for anti-M2 (KAN-1) titer: the extracellular part of M2 (KAN-1) (SLLTEVETPTRNEWECRCSDSSD) synthetic peptide (1.0 µg/ml), kindly provided by Suzanne Epstein at the Food and Drug Administration,
- NP (KAN-1) in 1∶3 dilution with PBS, isolated from the supernatant of NP (KAN-1) encoded plasmid transfected 293T cells.

End-point titers were determined by linear regression analysis of the absorbance values (OD 450) as previously described [32]–[34], with R^2^>0.9 obtained from a series of three-fold dilutions, as the cut-off value was set as 0.3.

### Microneutralization assay of mouse and ferret sera

A microneutralization assay to detect humoral neutralization responses against influenza was performed as previously described [22], [35]. For mice, two-fold dilutions of heat-inactivated sera were tested for the presence of antibodies that neutralized the infectivity of 100 TCID50 (50% tissue culture infectious dose) of H5N1 viruses on MDCK cell monolayers using two wells per dilution on a 96-well plate as described [35]. After 2 days of incubation, cells were fixed, and ELISA was performed to detect the presence of viral nucleoprotein (NP) and determine the neutralization activity. For ferrets, neutralizing antibody activity was analyzed in an MN assay based on the methods of the WHO Global Influenza Program [36]. Sera were treated with receptor-destroying enzyme by diluting one part serum with three parts enzyme and incubated overnight in a 37°C water bath and heat-inactivated as described for the HAI assay. Virus strains used for the MN assay are low-pathogenic, H5N1-PR8 re-assortants, obtained from Ruben Donis at the CDC Influenza Branch (Atlanta, GA): Clade 1, A/Vietnam/1203/2004(H5N1)/PR8-IBCDC-RG and Clade 2.1, A/Indo/5/2005(H5N1)/PR8-IBCDC-RG2. Seed stocks of the re-assortant strains were obtained and expanded at BIOQUAL in 10-day-old embryonated chicken eggs.

### Hemagglutination inhibition (HAI) assay of ferret sera

HAI assays were performed using four hemagglutinin units of virus and 1% horse RBC as previously described [37]–[40]. Ferret sera were treated with receptor-destroying enzyme by diluting one part serum with three parts enzyme and incubated overnight in a 37°C water bath. The enzyme was inactivated by 30 min. incubation at 56°C followed by addition of six parts PBS for a final dilution of 1/10. Virus strains used for the HAI assay were low-pathogenic, H5N1-PR8 re-assortants obtained from Ruben Donis at the CDC Influenza Branch (Atlanta, GA): Clade 1, A/Vietnam/1203/2004(H5N1)/PR8-IBCDC-RG and Clade 2.1, A/Indo/5/2005(H5N1)/PR8-IBCDC-RG2. Seed stocks of the re-assortant strains were obtained and expanded at BIOQUAL in 10-day-old embryonated chicken eggs. Virus strains used for the HAI assay were identical to the low pathogenic re-assortants listed for the MN assay.

### Production of pseudotyped lentiviral vectors and measurement of neutralizing antibodies from mouse and ferret serum

The recombinant lentiviral vectors expressing a luciferase reporter gene were produced as previously described [22], [29], [31], [41]. This assay has been developed as a safer, highly sensitive alternative to HAI and MN assays that can be applied in a high-throughput format for influenza vaccine evaluation [42]–[44]. Briefly, HA-pseudotyped lentiviral vectors encoding luciferase were first titrated by serial dilution. The concentration of virus giving 25% maximum activity was then incubated with the indicated amounts of mouse anti-serum before being added to 293A cells. Plates were washed and replaced with fresh media 14–16 hours later. Luciferase activity was measured after 48 hours as previously described [45] using mammalian cell lysis buffer and luciferase assay reagent (Promega, Madison, WI) according to the manufacturer's protocol.

### Statistical analysis

Linear regression was utilized to determine the end-point titers of the antibodies against different antigens. In addition, the survival differences between animal groups were tested by log-rank test using GraphPad Prism software (San Diego, CA). End-point antibody titers in log 10 scale of different groups were compared by one-way Analysis of Variance (ANOVA). If this was significant at alpha = .05, we proceeded to look at pairwise comparisons using t-tests. For four or more groups (mice studies), the *p*-values from the pairwise tests were compared to a Bonferroni-adjused threshold of .05/6 = .008, where 6 comparisons were being made between each set of 4 groups with relevant immunogens. For three groups (ferret studies), we used instead Fisher's Least Significant Difference method to maintain a threshold of alpha = .05 for the pairwise tests following a significant ANOVA.

## Results

### Immunogen expression in mammalian cells

**Figure 1:**
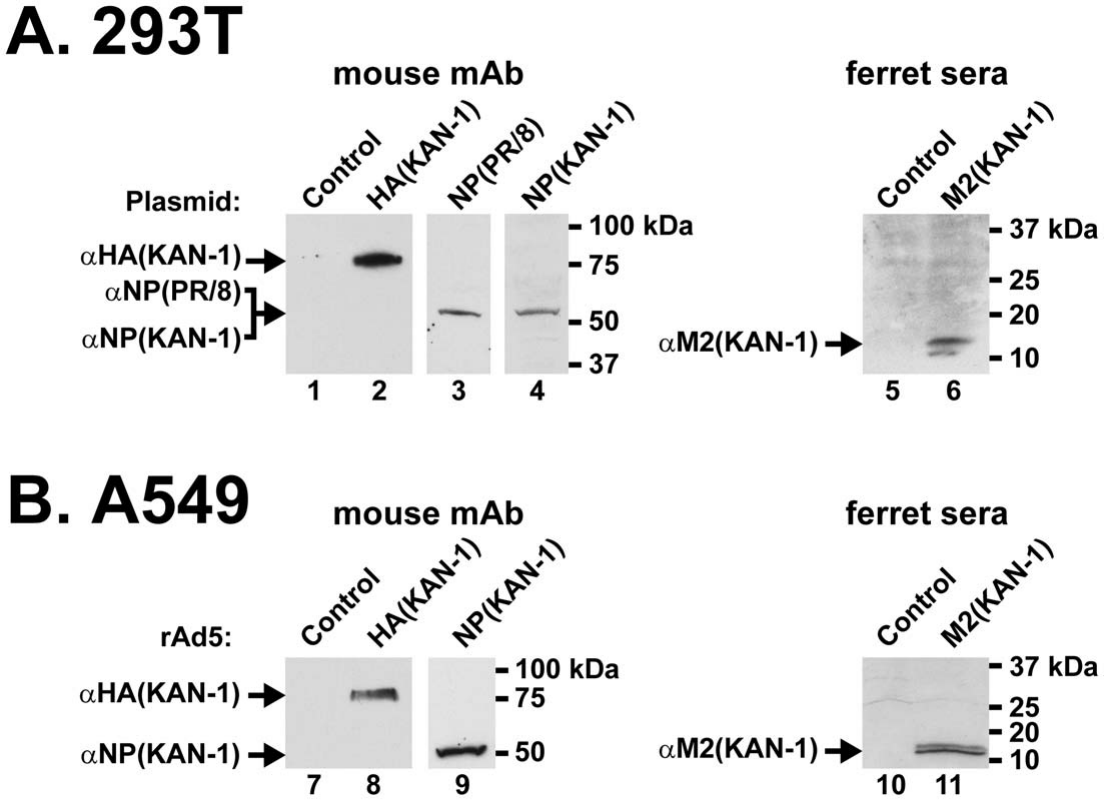
Expression of immunogens using DNA and rAd5 vectors in cell culture. (A) Western blot analysis confirmed the expression of HA protein of A/Thailand/1/KAN-1/2004 (lane 2), NP protein of A/PR/8/34 (lane 3) and A/Thailand/1/KAN-1/2004 (lane 4), and M2 protein of A/Thailand/1/KAN-1/2004 (lane 6) in 293T cells transfected by eukaryotic plasmid expression vectors. (B) Expression of rAd5 vectors was confirmed in A549 cells after transduction with vectors encoding HA (KAN-1) (lane 8), NP (KAN-1) (lane 9), and M2 (KAN-1) (lane 11). Arrows indicate the relevant predicted size of the indicated viral proteins. Bands refer to the right predicted size of different viral proteins that were detected in each lane as indicated. Molecular weight markers were used for protein size reference.

Prior to animal immunizations, expression of specific influenza viral genes was confirmed in 293T cells (Figure 1A) or A549 cells (Figure 1B). Western blot analysis confirmed the expression of HA protein of A/Thailand/1/KAN-1/2004 (Figure 1A, lane 2), NP protein of A/PR/8/34 (Figure 1A, lane 3) and A/Thailand/1/KAN-1/2004 (Figure 1A, lane 4), and M2 protein of A/Thailand/1/KAN-1/2004 (Figure 1A, lane 6) in 293T cells transfected by eukaryotic plasmid expression vectors. To confirm expression of rAd5 vectors, A549 cells were analyzed by Western blot analysis after transduction with vectors encoding HA (KAN-1) (Figure 1B, lane 8), NP (KAN-1) (Figure 1B, lane 9), and M2 (KAN-1) (Figure 1B, lane 11).

### Combinatorial DNA vaccination with HA, NP, and M2 followed by viral challenge in mice

**Figure 2:**
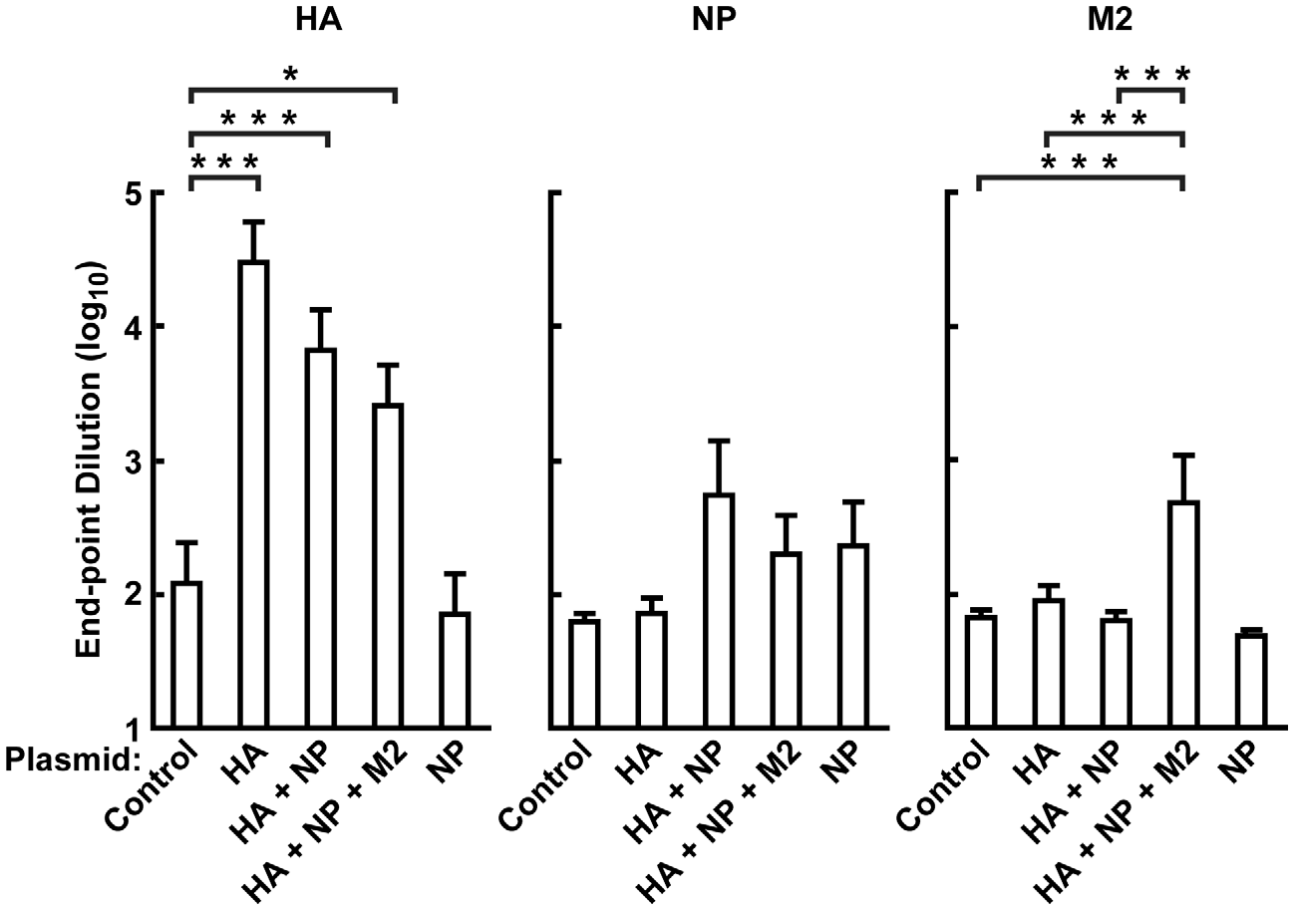
Detection of humoral immune responses to HA, NP and M2 by ELISA after DNA vaccination in mice. Sera from individual mice immunized with HA, HA+NP, HA+NP+M2, NP and vector control were collected 10 days after the third DNA immunization. The sera were subjected to ELISA assay to determine their end-point titers against HA(KAN-1), NP(PR8), and M2 (KAN-1). Each bar represents the group mean (*n* = 5 for NP, control; *n* = 10 for HA, HA+NP, HA+NP+M2) for the end-point titers of the total IgG and IgM against HA(KAN-1) purified protein (HA), against NP(PR/8) purified protein (NP), and against M2(KAN-1) extracellular domain peptide (M2), as indicated. Each immunized group was compared to controls as well as other groups containing relevant immunogens. ANOVA tests were significant for the responses against HA and M2, but not against NP. For HA and M2, significant pairs of groups are noted on the graph. Only *p*-values less than 0.05/6 = 0.0083 (Bonferroni Correction) were considered significant for these pairwise comparisons. A single asterisk (*) represents a *p*-value between 0.008 and 0.001, while ** indicates <0.001, and *** indicates <0.0001. All HA-containing groups showed significant antibody responses against HA compared to controls (*p*<0.0013), but did not differ significantly among themselves. Differences between NP-immunized groups were at the border of statistical significance by ANOVA (*p* = 0.0504), as was the comparison between HA+NP and HA groups (*p* = 0.008) when adjusted for multiple comparisons. The only M2-containing group, HA+NP+M2, elicited a significant humoral response against M2 protein compared to control (*p*<0.0001).

We evaluated different DNA immunogens [HA (KAN-1) alone; HA (KAN-1) with NP (PR/8); HA (KAN-1) with NP (PR/8) and M2 (KAN-1); or NP (PR/8)] for their ability to elicit protective immunity against A/Vietnam/1203/2004 (H5N1) using the mouse challenge model. Study cohorts consisted of 10 female BALB/c mice for the HA alone, HA+NP, and HA+NP+M2 groups, and 5 animals for the NP alone and vector control groups. Serum was collected 10 days after the last DNA vaccination, and end-point ELISA titers were evaluated after DNA immunization (Figure 2). All HA-containing groups showed an increase in HA ELISA titer compared to the control cohort (*p*<0.0013) (Figure 2, left panel). As might be expected, these levels appeared to decrease with the number of gene products in the DNA vaccine, although significant differences were not observed amongst the HA-containing groups.

In analyzing antibody responses against NP protein (Figure 2, middle panel), the result of the ANOVA was a borderline *p*-value of 0.0504. Pairwise comparisons between the HA alone and HA+NP group were similar (*p* = .0080 with a Bonferroni-adjusted threshold of .05/6 = .0083). However, when analyzing antibody responses against M2 protein, DNA vaccination with M2 in combination with both HA and NP elicited a significant humoral response compared to controls (*p*<0.0001) (Figure 2, right panel). This result suggests that M2 is immunogenic, although immunization with M2 alone was not included in this study.

To define the neutralizing antibody responses, mouse sera were pooled by groups and pseudotype neutralization, and microneutralization assays were performed. These analyses revealed neutralization activity in the HA group, with lower titers in animals immunized with HA+NP and lowest responses in those vaccinated with HA+NP+M2 (Table 1). However, minimal neutralization was evident in NP-only immunized animals. HAI assays were not performed on the mouse sera.

**Table 1:** Neutralizing Antibody Responses of HA-Vaccinated Mice and Ferrets.

Animal	Immunogen	Lentiviral Inhibition (IC50)	HAI titer	MN titer
**Mice**				
37	Control vector	0	NA	<20
31	HA	382	NA	80
32	HA+NP	151	NA	40
33	HA+NP+M2	<100	NA	20
34	NP	0	NA	30
**Ferrets**				
1	Control vector	0	<20	<20
2	Control vector	0	<20	<20
3	Control vector	0	<20	<20
4	Control vector	0	<20	<20
5	HA	5691	NA	NA
6	HA	1353	1280	640
7	HA	1239	160	80
8	HA	4636	1280	640
9	HA+NP+M2	2047	640	320
10	HA+NP+M2	826	640	160
11	HA+NP+M2	4621	1280	320
12	HA+NP+M2	2466	1280	320
13	Control vector	UD	<20	<20
14	Control vector	UD	<20	<20
15	Control vector	UD	<20	<20
16	Control vector	UD	<20	<20
17	Control vector	UD	<20	<20
18	NP	UD	<20	<20
19	NP	UD	<20	<20
20	NP	UD	<20	<20
21	NP	UD	<20	<20
22	NP+M2	UD	<20	<20
23	NP+M2	UD	<20	<20
24	NP+M2	UD	<20	<20
25	NP+M2	UD	<20	<20
26	NP+M2	UD	<20	<20
27	M2	UD	<20	<20
28	M2	UD	<20	<20
29	M2	UD	<20	<20
30	M2	UD	<20	<20

*UD = Undetectable; NA = Not Assessed.

Neutralization was determined by lentiviral inhibition assay, hemagglutinin inhibition assay, and microneutralization assay. Sera from the indicated mouse and ferret immunizations with the indicated viral antigens by DNA alone or DNA/rAd5 before the viral challenge were evaluated by pseudotyped lentiviral inhibition, hemagglutinin inhibition (HAI), and microneutralization assays (MN). UD represents serum samples with undetectable neutralization activities even at the lowest dilutions, while NA represents samples that were not available, and therefore not assessed. In both mice and ferrets, only HA-containing groups stimulated strong humoral responses.

**Figure 3:**
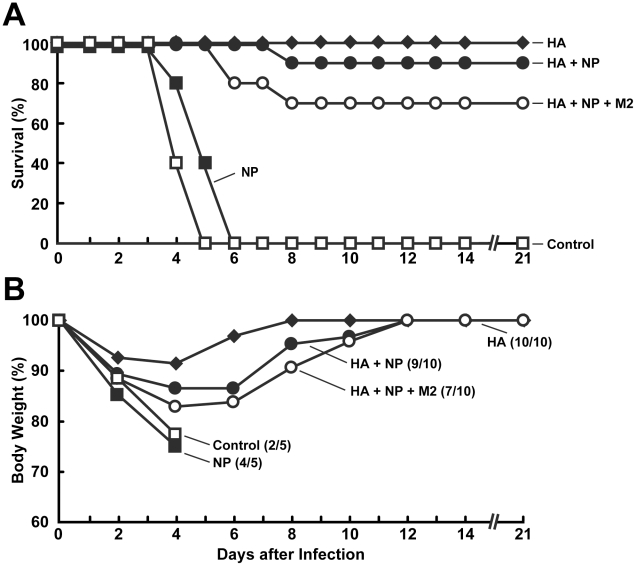
DNA immunization with HA, HA+NP, and HA+NP+M2 induces similar protection after A/Vietnam/1203/2004 virus challenge in mice. (A) Survival data is shown as a percentage comparing the final animal number at day 21 with the initial animal number in each group. All HA-containing groups showed significant survival compared to controls. There is no statistical difference between the HA, HA+NP and HA+NP+M2 groups (*p* = 0.317 between HA and HA+NP; *p* = 0.146 between HA and HA+NP+M2; *p* = 0.515 between HA+NP and HA+NP+M2 by log-rank test); NP and the control groups were not statistically different from each other. (B) Body weights of the mice were also monitored and the total body weight of all of the surviving animals in each group was compared with the respective initial body weights. As expected, the HA group showed the least amount of body weight loss, with the other HA-containing groups showing similar patterns. However, the NP-immunized group demonstrated severe weight loss, similar to controls.

To determine the efficacy of these alternative DNA immunizations, mice were challenged with the HPAI A/Vietnam/1203/2004 virus. Mice were challenged intranasally with 100 LD_50_ of A/Vietnam/1203/2004 virus, providing a stringent evaluation of protective efficacy. All animals in the control and NP alone groups died within 6 days after viral challenge, whereas animals immunized with HA alone, HA+NP and HA+NP+M2 showed survival rates of 100, 90 and 70%, respectively (Figure 3A); these survival rates were not statistically significantly different. As expected, the HA alone group showed the least amount of body weight loss, while groups HA+NP and HA+NP+M2 showed similar weight loss patterns (Figure 3B). In contrast, the control and NP groups that showed no immune protection demonstrated severe weight loss (Figure 3B). This finding suggests that NP DNA immunization does not confer protection against H5N1 viral challenge at the doses used here.

### Comparative efficacy of DNA/rAd5 vaccination with different combinations of HA, NP and M2 in ferrets

**Figure 4:**
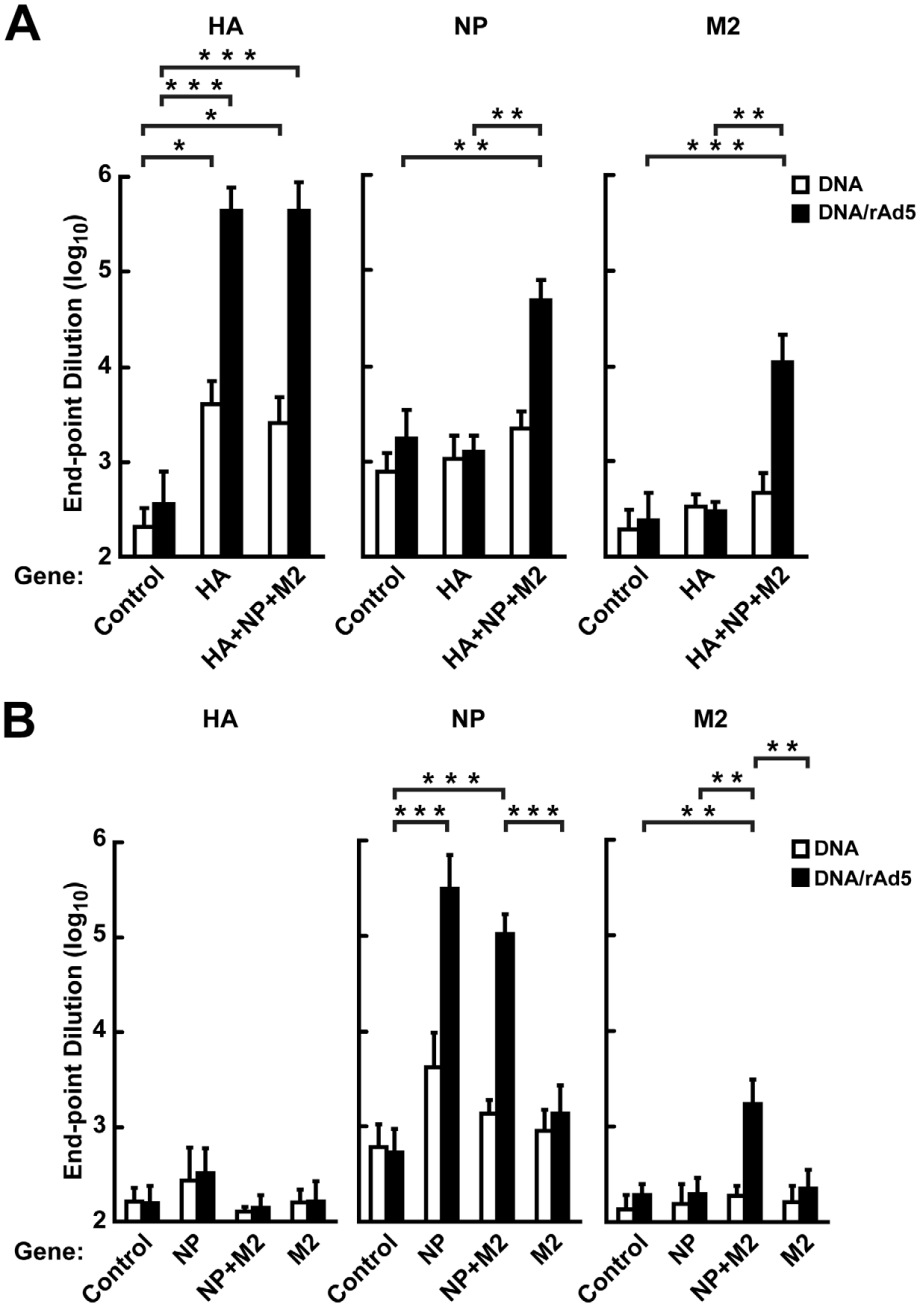
Humoral immune responses to HA, NP and M2 confirmed by ELISA after DNA/rAd5 immunization in ferrets. (A) Sera from the HA, HA+NP+M2 or vector control immunized ferrets were collected 14 days after the third DNA immunization (white bars), and 14 days after the recombinant adenovirus boost (solid bars), and subjected to ELISA assays to determine their end-point titer levels against HA(KAN-1), NP(KAN-1), and M2(KAN-1) antigens. Each bar represents the group mean for the end-point titers of total IgG and IgM, determined in duplicate by series dilution of ELISA assay with the error bars indicating the standard deviation. ANOVA tests were significant for only the responses against HA at the first time point, and for all three antigens after the rAd5 boost. For HA and M2, significant pairs of groups are noted on the graph. Only *p*-values less than 0.05 are indicated. * represents a *p*-value between 0.05 and 0.001, while ** indicates <0.001, and *** indicates <0.0001. As expected, the HA alone group elicited significant anti-HA immunity that increased after rAd5 HA boost (left panel) compared to controls (*p*<0.0001). The HA+NP+M2 group elicited similar anti-HA ELISA antibodies, as well as significant anti-NP humoral responses (*p* = 0.0006) and anti-M2 responses (*p*<0.0001) after the rAd boost (middle and right panels). (B) Sera from the NP, M2, NP+M2 or vector controls were collected 14 days after the third DNA immunization (white bars), and the sera from the same animals were also collected 14 days after the recombinant adenovirus boost (solid bars). ANOVA tests were not significant for any of the antigens at the first time point, and for NP and M2 after the rAd5 boost. For NP and M2, significant pairs of groups are noted on the graph. Only *p*-values less than 0.008 are indicated. * represents a *p*-value between 0.008 and 0.001, while ** indicates <0.001, and *** indicates <0.0001. For both NP and NP+M2 immunized groups, significant anti-NP humoral responses were observed after the rAd boost (*p*<0.0001) (middle panel). Significant anti-M2 humoral responses were detected in animals immunized with NP+M2 post-rAd boost (*p* = 0.0005), but not in the M2 alone group (right panel).

To determine the comparative efficacy of alternative gene-based vaccines in ferrets, we immunized ferrets with these gene products in different combinations in a triple DNA inmmunization. In this experiment, animals received a recombinant adenovirus serotype 5 (rAd5) boost in order to increase the immunogenicity of DNA priming. Ferrets were immunized with HA alone (*n* = 4); HA+NP+M2 (*n* = 4); NP alone (*n* = 4); NP+M2 (*n* = 5); M2 alone (*n* = 4); or empty vector controls (*n* = 4+5). ELISA titers to HA, NP and M2 were determined after DNA immunization (Figure 4, white bars) and after the rAd5 boost (Figure 4, black bars). As expected, after DNA immunization, the HA alone group elicited significant anti-HA immunity that increased after rAd5 HA boost (Figure 4A, left panel) compared to controls (*p*<0.0001). The HA+NP+M2 group elicited similar anti-HA ELISA antibodies, as well as significant anti-NP humoral responses (*p* = 0.0006) and anti-M2 responses (*p*<0.0001) after the rAd boost (Figure 4A, middle and right panels) when compared to controls. For both NP and NP+M2 immunized groups, significant anti-NP humoral responses were observed after the rAd boost (*p*<0.0001) (Figure 4B, middle panel) compared to controls. Significant anti-M2 humoral responses were detected in animals immunized with NP+M2 post-rAd boost (*p* = 0.0005), but not in the M2 alone group (Figure 4B, right panel) relative to controls. Anti-M2 humoral responses were not detectable in most cases, except after DNA/rAd5 immunization with M2 in combination with NP.

The ability of the HA antibodies from immunized ferrets to neutralize H5N1 virus was analyzed with three assays: a pseudotyped lentiviral vector neutralizing assay, an HA inhibition assay, and a microneutralization assay (Table 1; Ferrets). Only HA-containing groups showed substantial neutralizing antibody titer responses, while NP, M2, and NP+M2 groups showed no neutralization in each assay.

**Figure 5:**
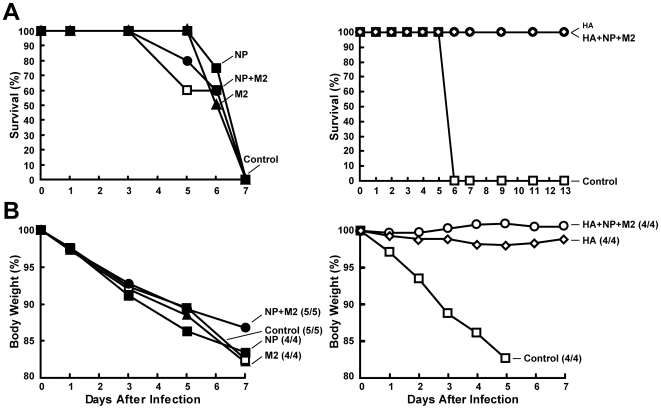
Protection of DNA/rAd5 vaccines encoding HA or HA+NP+M2, but not NP, NP+M2, or M2, against A/Vietnam/1203/2004 virus challenge. (A) Ferrets immunized three times with DNA followed by a single rAd5 boost were challenged under anesthesia with 10^7^ EID_50_/ferret of influenza virus A/Vietnam/1203/2004. The animals were monitored 7 days for survival, shown as a percentage comparing the initial animal number to the final animal number in the same group (left panel). There was no statistical difference between the control group and groups immunized with NP, NP+M2, or M2. Both the HA and HA+NP+M2 groups showed 100% survival (right panel), whereas the vector control group showed 0% survival after the viral challenge. There was no statistically significant difference between the HA and HA+NP+M2 groups (*p* = 1.00), but there was a significant difference between these groups and the control (*p* = 0.008), by a log-rank test. (B) Body weights of the ferrets were also monitored and the total body weight of all of the surviving animals in each group was compared with the respective initial body weight (left panel). Ferrets immunized with HA and HA+NP+M2 groups showed no weight loss, while the control group ferrets showed rapid weight loss (right panel). The survival and initial animal numbers in each group on the last day of body weight data collection are indicated next to the curve labels. The survival percentage for each group was analyzed statistically by a log-rank test.

At least nine weeks after the rAd5 boost, ferrets were challenged with a high dose of A/Vietnam/1203/2004 (H5N1) virus. All groups that lacked HA, including NP alone, NP+M2, and M2 alone, developed severe disease, manifested by significant weight loss and neurological dysfunction less than seven days after the viral challenge, similar to the clinical symptoms observed in the control group (Figure 5A, left panel). The animals were euthanized due to severity of symptoms. The body weight loss among these groups was very similar (Figure 5B, left panel). In contrast, the HA and HA+NP+M2 groups were completely protected from lethality after a high dose of influenza virus challenge compared to no survival in empty vector immunized controls (Figure 5A, right panel). Three animals showed very mild clinical signs such as slight temperature elevation or weight loss three days after the viral challenge, but these symptoms disappeared within two days. Body weight of the two groups remained steady post-viral challenge in HA-immunized ferrets (Figure 5B, right panel), in contrast to the controls. Viral titers from the nasal washes confirmed the antiviral effects of HA- but not NP-containing vaccines (data not shown). Control animals showed peak viral titers at day 3, and subsequently died by day 6. Both HA and HA+NP+M2 groups showed moderate viral titers at day 3, followed by quick clearance with no viral titers detectable at day 5 and day 7 (data not shown). Survival, body weight, and viral titers are indistinguishable between the HA and HA+NP+M2 groups after viral challenge, suggesting that the NP and M2 did not contribute to immune protection in ferrets. HA immune responses alone were necessary and sufficient to protect ferrets from the lethal effects of infection under these challenge conditions.

## Discussion

The highly conserved viral genes NP and M2 have become a focus for the development of broad, cross-protective or “universal” influenza vaccines. In mice, several studies have shown that gene-based immunization with NP and M2 induce strong humoral and cellular responses, and protect against lethal H5N1 challenges [1], [4], [6], [9], [22], [35], [46]–[48]. In this study, mice were immunized with DNA vaccines encoding HA alone, NP alone, HA+NP, and HA+NP+M2. All HA-containing groups and the M2-containing group generated significant antibody responses against HA and M2 proteins, respectively. However, only the HA+NP group elicited a response against NP protein, and that was marginally significant at best. Moreover, while all HA-containing groups were protected against lethal H5N1 challenge with a survival rate of at least 70%, immunization with NP alone did not protect mice from lethal H5N1 challenge (Figure 3).

Previous studies with NP immunization have yielded mixed results, likely dependent on the mode of gene-based vaccination and on the dose of challenge virus. Though the first studies using NP DNA immunization conferred protection against lethal influenza challenge [5], [48], in retrospect, this result may be seen with relatively low challenge doses. In a more recent study, a challenge dose that overcame NP DNA vaccine protection was similar to the amount used here [2]. NP immunization with DNA/rAd derived from H1N1 strain A/PR/8 resulted in protection in mice against challenge with a heterologous virus strain, H5N1 A/HK/156, but no protection was seen against a more virulent strain, A/HK/483 [1]. It appears that protection afforded by immunization with NP in mice diminishes markedly as the dose and virulence of the challenge virus increase. However, DNA vaccination with NP in combination with M2 has been shown to protect mice in both Vaxfectin formulations and rAd-boost regimens [7], [20], [21]. While we did not investigate M2 alone in mice, a previous study has shown that vaccination with M2 alone is capable of protecting mice against heterologous strains of influenza virus challenge, including H5N1 [9].

Despite showing no neutralizing antibody responses, mice immunized with HA+NP+M2 were fully protected against lethal challenge. Lalor et al. showed a similar result in which a Vaxfectin-formulated DNA vaccine encoding H5+NP+M2 consensus genes protected mice against H5N1 challenge, despite low HAI responses. This is suggestive of other useful mechanisms of protection, such as cytotoxic T-lymphocytes, although this was not assessed in our study. A previous study with DNA/rAd5 immunization has also suggested that cellular immunity may contribute to protection in this model [12].

While the mouse model has been used for immunogenicity studies, the murine disease does not have the same pathogenicity as human infection and is not ideal for H5N1 infection studies. These differences may be due to species changes in HA receptor specificity and distribution, as well as differential immunopathogenicity [13]–[17]. In contrast, it is generally accepted that ferrets exhibit pathology more similar to humans after H5N1 infection, including severe lethargy, fever, weight loss, transient lymphopenia, and viral replication in the upper/lower respiratory tracts and multiple systemic organs [13]. Furthermore, human isolates of influenza virus have been shown to attach and infect ferret airways [49], [50], indicating that humans and ferrets share similar HA receptor specificity [51].

Ferrets were immunized with HA alone, NP alone, M2 alone, NP+M2, HA+NP+M2, and control using gene-based vaccines delivered in DNA/rAd5 vectors. Since there are no established assays available to measure cellular responses against HA, NP and M2 in ferrets, the efficacy of these immunogens was evaluated based on the measurement of neutralizing antibody titers through microneutralization and HA-inhibition assays. When HA and NP were present, significant humoral responses were stimulated against these proteins. However, M2 antibodies were only stimulated when M2 was in combination with NP, suggesting possible immune synergy of the two gene products. Similar adjuvant effects of NP in the anti-M2 response have recently been reported in mice [21]. Each group was challenged with a lethal strain of HPAI H5N1, and only vaccines containing HA conferred protection while NP, M2, and NP+M2 groups did not survive.

Lalor et al. showed NP+M2 to be protective in mice, but only when formulated with Vaxfectin and in a vaccine dose 6.6 times greater than that used in this study. However, in ferrets, in the absence of HA, this combination only resulted in delayed illness and death [20]. Price et al. showed that NP+M2 protects ferrets against H5N1 in a triple-prime rAd boost regimen similar to the one used here, but this difference in results may be due to the higher dose of DNA in the primary immunization (ten-fold greater than the present study) as well as a much lower viral challenge dose (5×LD_50_ [21] compared to 3×10^5^×LD_50_ in this study). However, due to differences in experimental parameters such as the immunization regimen and assay standardization, direct comparisons of NP and M2 immune responses between studies are difficult. In addition, different vaccinations may alter various antibody and cellular immune responses which may affect the protective immunity in various animal models. Nonetheless, the evidence suggests while NP or NP+M2 may provide moderate levels of protection against low dose viral challenges in ferrets, they are insufficient against high challenge doses of HPAI. On the other hand, HA elicits effective immune protection even against very high HPAI viral challenge doses. Although vaccines encoding NP or M2 alone are not required for protection against H5N1, they could potentially augment HA-encoded vaccines, particularly when there is a mismatch between the vaccine and viral HA proteins. Studies in mice have shown that M2 antibodies may help to reduce viral replication [8], [9], [52], [53], while studies in ferrets have shown M2 to be associated with reductions in viral recovery [20], [54]. However, based on our H5N1 challenge results in ferrets, HA DNA immunization is superior to NP and M2 DNA immunization in terms of protection. These highly conserved viral genes may require combinatorial vaccination with HA to be suitable candidates for universal influenza vaccines.

## References

[1] Epstein SL, Kong WP, Misplon JA, Lo CY, Tumpey TM (2005). Protection against multiple influenza A subtypes by vaccination with highly conserved nucleoprotein.. Vaccine.

[2] Patel A, Tran K, Gray M, Li Y, Ao Z (2009). Evaluation of conserved and variable influenza antigens for immunization against different isolates of H5N1 viruses.. Vaccine.

[3] Robinson HL, Boyle CA, Feltquate DM, Morin MJ, Santoro JC (1997). DNA immunization for influenza virus: studies using hemagglutinin- and nucleoprotein-expressing DNAs.. J Infect Dis.

[4] Ulmer JB, Fu TM, Deck RR, Friedman A, Guan L (1998). Protective CD4+ and CD8+ T cells against influenza virus induced by vaccination with nucleoprotein DNA.. J Virol.

[5] Yankauckas MA, Morrow JE, Parker SE, Abai A, Rhodes GH (1993). Long-term anti-nucleoprotein cellular and humoral immunity is induced by intramuscular injection of plasmid DNA containing NP gene.. DNA Cell Biol.

[6] Ernst WA, Kim HJ, Tumpey TM, Jansen AD, Tai W (2006). Protection against H1, H5, H6 and H9 influenza A infection with liposomal matrix 2 epitope vaccines.. Vaccine.

[7] Jimenez GS, Planchon R, Wei Q, Rusalov D, Geall A (2007). Vaxfectin-formulated influenza DNA vaccines encoding NP and M2 viral proteins protect mice against lethal viral challenge.. Hum Vaccin.

[8] Neirynck S, Deroo T, Saelens X, Vanlandschoot P, Jou WM (1999). A universal influenza A vaccine based on the extracellular domain of the M2 protein.. Nat Med.

[9] Tompkins SM, Zhao Z-S, Lo C-Y, Misplon JA, Liu T (2007). Matrix protein 2 vaccination and protection against influenza viruses, including subtype H5N1.. Emerg Infect Dis.

[10] Portela A, Digard P (2002). The influenza virus nucleoprotein: a multifunctional RNA-binding protein pivotal to virus replication.. J Gen Virol.

[11] Pinto LH, Lamb RA (2007). Controlling influenza virus replication by inhibiting its proton channel.. Mol Biosyst.

[12] Lo C-Y, Wu Z, Misplon JA, Price GE, Pappas C (2008). Comparison of vaccines for induction of heterosubtypic immunity to influenza A virus: Cold-adapted vaccine versus DNA prime-adenovirus boost strategies.. Vaccine.

[13] Zitzow LA, Rowe T, Morken T, Shieh W-J, Zaki S (2002). Pathogenesis of avian influenza A (H5N1) viruses in ferrets.. J Virol.

[14] Ibricevic A, Pekosz A, Walter MJ, Newby C, Battaile JT (2006). Influenza virus receptor specificity and cell tropism in mouse and human airway epithelial cells.. J Virol.

[15] Rogers GN, Paulson JC (1983). Receptor determinants of human and animal influenza virus isolates: differences in receptor specificity of the H3 hemagglutinin based on species of origin.. Virology.

[16] Lee CW, Suarez DL, Tumpey TM, Sung HW, Kwon YK (2005). Characterization of highly pathogenic H5N1 avian influenza A viruses isolated from South Korea.. J Virol.

[17] Lu X, Tumpey TM, Morken T, Zaki SR, Cox NJ (1999). A mouse model for the evaluation of pathogenesis and immunity to influenza A (H5N1) viruses isolated from humans.. J Virol.

[18] Belser JA, Lu X, Maines TR, Smith C, Li Y (2007). Pathogenesis of avian influenza (H7) virus infection in mice and ferrets: enhanced virulence of Eurasian H7N7 viruses isolated from humans.. J Virol.

[19] Maines TR, Lu XH, Erb SM, Edwards L, Guarner J (2005). Avian influenza (H5N1) viruses isolated from humans in Asia in 2004 exhibit increased virulence in mammals.. J Virol.

[20] Lalor PA, Webby RJ, Morrow J, Rusalov D, Kaslow DC (2008). Plasmid DNA-based vaccines protect mice and ferrets against lethal challenge with A/Vietnam/1203/04 (H5N1) influenza virus.. J Infect Dis.

[21] Price GE, Soboleski MR, Lo CY, Misplon JA, Pappas C (2009). Vaccination focusing immunity on conserved antigens protects mice and ferrets against virulent H1N1 and H5N1 influenza A viruses.. Vaccine.

[22] Kong W-P, Hood C, Yang Z-Y, Wei CJ, Xu L (2006). Protective immunity to lethal challenge of the 1918 pandemic influenza virus by vaccination.. Proc Natl Acad Sci USA.

[23] Catanzaro AT, Roederer M, Koup RA, Bailer RT, Enama ME (2007). Phase I clinical evaluation of a six-plasmid multiclade HIV-1 DNA candidate vaccine.. Vaccine.

[24] Rasmussen H, Rasmussen C, Lempicki M, Durham R, Brough D (2002). TNFerade Biologic: Preclinical toxicology of a novel adenovector with a radiation-inducible promoter, carrying the human tumor necrosis factor alpha gene.. Cancer Gene Ther.

[25] Brough DE, Lizonova A, Hsu C, Kulesa VA, Kovesdi I (1996). A gene transfer vector-cell line system for complete functional complementation of adenovirus early regions E1 and E4.. J Virol.

[26] Butman BT, Lizonova A, Brough DE, Sowers JM, Sheets R, Petricciani J, Sheets R (2006). Comprehensive characterization of the 293-ORF6 cell line.. Vaccine cell substrates 2004.

[27] Reed LJ, Muench H (1938). A simple method for estimating fifty percent endpoints.. Am J Hyg.

[28] Richmond JY, McKinney RW, Richmond JY, McKinney RW (1999). Laboratory Biosafety Level Criteria.. Biosafety in Microbiological and Biomedical Laboratories.

[29] Yang Z-Y, Kong W-P, Huang Y, Roberts A, Murphy B (2004). A DNA vaccine induces SARS coronavirus neutralization and protective immunity in mice.. Nature.

[30] Rao S, Kong WP, Wei CJ, Yang ZY, Nason M (2008). Multivalent HA DNA vaccination protects against highly pathogenic H5N1 avian influenza infection in chickens and mice.. PLoS ONE.

[31] Wei CJ, Xu L, Kong WP, Shi W, Canis K (2008). Comparative efficacy of neutralizing antibodies elicited by recombinant hemagglutinin proteins from avian H5N1 influenza virus.. J Virol.

[32] Craft JE, Grodzicki RL, Steere AC (1984). Antibody response in Lyme disease: evaluation of diagnostic tests.. J Infect Dis.

[33] Angarano G, Laddago V, Materi AM (1984). Comparison of three enzyme-linked immunosorbent assays suitable for the detection of antibodies to rotaviruses in epidemiological studies.. Eur J Clin Microbiol.

[34] Graham DA, Mawhinney KA, McShane J, Connor TJ, Adair BM (1997). Standardization of enzyme-linked immunosorbent assays (ELISAs) for quantitative estimation of antibodies specific for infectious bovine rhinotracheitis virus, respiratory syncytial virus, parainfluenza-3 virus, and bovine viral diarrhea virus.. J Vet Diagn Invest.

[35] Rowe T, Abernathy RA, Hu-Primmer J, Thompson WW, Lu X (1999). Detection of antibody to avian influenza A (H5N1) virus in human serum by using a combination of serologic assays.. J Clin Microbiol.

[36] World Health Organization (2009). WHO Manual on Animal Influenza Diagnosis and Surveillance.

[37] Stephenson I, Wood JM, Nicholson KG, Charlett A, Zambon MC (2004). Detection of anti-H5 responses in human sera by HI using horse erythrocytes following MF59-adjuvanted influenza A/Duck/Singapore/97 vaccine.. Virus Res.

[38] Khurana S, Suguitan AL, Rivera Y, Simmons CP, Lanzavecchia A (2009). Antigenic fingerprinting of H5N1 avian influenza using convalescent sera and monoclonal antibodies reveals potential vaccine and diagnostic targets.. PLoS Med.

[39] Kang SM, Yoo DG, Lipatov AS, Song JM, Davis CT (2009). Induction of long-term protective immune responses by influenza H5N1 virus-like particles.. PLoS ONE.

[40] Suguitan AL, McAuliffe J, Mills KL, Jin H, Duke G (2006). Live, attenuated influenza A H5N1 candidate vaccines provide broad cross-protection in mice and ferrets.. PLoS Med.

[41] Yang Z-Y, Wei CJ, Kong W-P, Wu L, Xu L (2007). Immunization by avian H5 influenza hemagglutinin mutants with altered receptor binding specificity.. Science.

[42] Wang W, Butler EN, Veguilla V, Vassell R, Thomas JT (2008). Establishment of retroviral pseudotypes with influenza hemagglutinins from H1, H3, and H5 subtypes for sensitive and specific detection of neutralizing antibodies.. J Virol Methods.

[43] Nefkens I, Garcia JM, Ling CS, Lagarde N, Nicholls J (2007). Hemagglutinin pseudotyped lentiviral particles: characterization of a new method for avian H5N1 influenza sero-diagnosis.. J Clin Virol.

[44] Temperton N, Hoschler K, Major D, Nicolson C, Manvell R (2007). A sensitive retroviral pseudotype assay for influenza H5N1-neutralizing antibodies.. Influenza Other Respi Viruses.

[45] Yang Z-Y, Huang Y, Ganesh L, Leung K, Kong W-P (2004). pH-dependent entry of Severe Acute Respiratory Syndrome coronavirus is mediated by the Spike glycoprotein and enhanced by dendritic cell transfer through DC-SIGN.. J Virol.

[46] De Filette M, Min Jou W, Birkette A, Lyons K, Schultz B (2005). Universal influenza A vaccine: optimization of M2-based constructs.. Virology.

[47] Epstein SL, Stack A, Misplon JA, Lo CY, Mostowski H (2000). Vaccination with DNA encoding internal proteins of influenza virus does not require CD8(+) cytotoxic T lymphocytes: either CD4(+) or CD8(+) T cells can promote survival and recovery after challenge.. Int Immunol.

[48] Ulmer JB, Donnelly JJ, Parker SE, Rhodes GH, Felgner PL (1993). Heterologous protection against influenza by injection of DNA encoding a viral protein.. Science.

[49] Piazza FM, Carson JL, Hu SC, Leigh MW (1991). Attachment of influenza A virus to ferret tracheal epithelium at different maturational stages.. Am J Respir Cell Mol Biol.

[50] Hoke CH, Hopkins JA, Meiklejohn G, Mostow SR (1979). Comparison of sevral wild-type influenza viruses in the ferret tracheal organ culture system.. Rev Infect Dis.

[51] Leigh MW, Connor RJ, Kelm S, Baum LG, Paulson JC (1995). Receptor specificity of influenza virus influences severity of illness in ferrets.. Vaccine.

[52] Jegerlehner A, Schmitz N, Storni T, Bachmann MF (2004). Influenza A vaccine based on the extracellular domain of M2: weak protection mediated via antibody-dependent NK cell activity.. J Immunol.

[53] Treanor JJ, Tierney EL, Zebedee SL, Lamb RA, Murphy BR (1990). Passively transferred monoclonal antibody to the M2 protein inhibits influenza A virus replication in mice.. J Virol.

[54] Fan J, Liang X, Horton MS, Perry HC, Citron MP (2004). Preclinical study of influenza virus A M2 peptide conjugate vaccines in mice, ferrets, and rhesus monkeys.. Vaccine.

